# Light-Induced Enhancement of Cross-Linked Viologen-Fluorene Polymer Electrode for High-Performance Supercapacitors

**DOI:** 10.1021/acsomega.5c09977

**Published:** 2025-12-19

**Authors:** Sinem Altınışık

**Affiliations:** Department of Chemical Engineering, 52950 Çanakkale Onsekiz Mart University, 17100 Çanakkale, Türkiye

## Abstract

Porous organic polymers (POPs) are generally prepared as insoluble powders and coated onto the electrode surface with another polymer binders such as PVDF: HFP during electrode preparation. POP-based electrodes formed through surface cross-linking hold promise for supercapacitor applications. In this study, a new pyridinium-fluorene donor–acceptor monomer (FBP_allyl) was synthesized and cross-linked onto graphene sheet conductive substrates via a thiol–ene click reaction to prepare a photoactive polymer electrode with light-harvesting and charge storage capability. The specific capacitance of the fabricated electrodes at 2.0 A/g was almost doubled under visible light, from 152.7 F/g (dark) to 304.1 F/g (light). In a three-electrode configuration, the electrochemical cell exhibited a power density of over 3000 W/kg, while the energy density increased from 35 to 60 Wh/kg under illumination. Furthermore, the prepared electrode cell showed long-term stability over 10,000 cycles, demonstrating that combining donor–acceptor engineering with thiol–ene cross-coupling is an effective approach for developing photoassisted capacitors with improved electrochemical performance.

## Graphical abstract

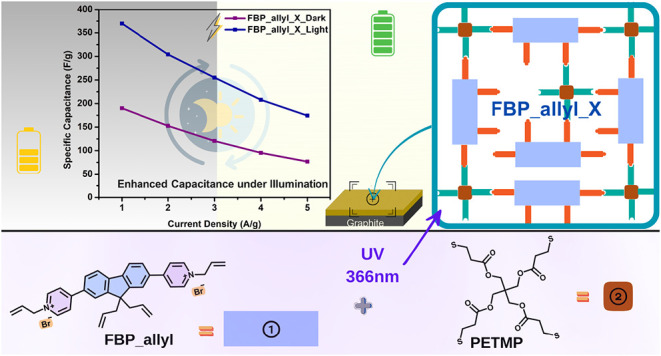

## Introduction

Electrochemical capacitors, combining high power and acceptable energy density, offer a versatile solution for energy storage applications.^1,2^ Among these, supercapacitors (SCs) stand out with their high-power density, fast charge–discharge capability and long cycle stability.^3–5^ Energy can be stored in SCs via capacitive or pseudocapacitive mechanisms. The capacitive (non-Faradaic) process relies on charge separation at the electrode/electrolyte interface, while the pseudocapacitive (Faradaic) process relies on redox reactions occurring in the electrode materials.^6–10^ Photoelectrochemical capacitors, built on these principles, have emerged as integrated energy devices that can combine electrochemical charge storage with other devices in a single system using advanced light-harvesting materials.^11,12^ When these systems are exposed to light, photogenerated electron–hole pairs initially separate within the semiconductor; electrons then migrate to the bulk and are stored at the electrode–electrolyte interface via ion adsorption, while holes participate in interfacial redox reactions with electrolyte species.^13^ In pioneering studies, semiconductors such as TiO_2_, Fe_2_O_3_ and BiVO_4_ and pseudocapacitive materials such as RuO_2_, MnO_2_ and Ni­(OH)_2_ have been extensively designed to form heterojunction structures to improve critical parameters such as charge separation and storage capacity along with the improvement of light absorption.^14,15^ This strategy can significantly improve device performance, but it can cause several fundamental problems, including limited ion adsorption capacity due to the nature of pristine semiconductors, recombination of photogenerated carriers, and side reactions that reduce stability·.^16^ To overcome these limitations, the design of multifunctional electrode materials with suitable band gaps, efficient charge separation pathways, and strong electrochemical stability is crucial. This strategy paves the way for the development of next generation photoelectrochemical capacitors with efficient light energy harvesting and high charge storage capacity.^17^

Electroactive POPs possess diverse charge transport (electron, ion, hole) behaviors due to their switchable conjugated backbones, stacked layers, and open junctions.^18^ These properties make them strong candidates for electrochemical energy storage and conversion applications.^19^ However, the electrode preparation procedure is crucial for improving the performance of POP-based electrodes. One of the innovative methods in this field is the thiol–ene click reaction, which, due to its simplicity, efficiency, and versatility, can enable the cross-linking of electroactive polymers directly at the electrode surface.^20,21^ This photoinduced process provides control over polymer network formation on the electrode surface, as well as high conversion efficiency and homogeneity of the resulting films.^22^ Thanks to these properties, stable polymer coatings can be produced that ensure long-term operation of electrochemical devices.^23^ Recent studies have shown that polymer networks produced by thiol–ene click chemistry exhibit high ionic conductivity and improved cycling durability at the electrolyte or electrode interface in energy storage systems.^24^ Furthermore, the integration of this method with conjugated donor–acceptor building blocks enables the fabrication of multifunctional electrodes for electrochemical supercapacitors.^25^

In summary, thiol–ene click reactions are quite useful for producing photopatterned and cross-linked fluorene-based polymer films on substrates such as glass and silicon.^22,26^ Recently, viologen based materials, which are effective n-type semiconductors due to their reversible redox behavior in the cathodic region, have been integrated into polymer matrices by this method for many applications.^27^ In addition, cross-linked polymer networks produced by combining donor–acceptor moieties offer a promising path to next-generation energy storage devices as well as optoelectronic systems. Herein, a fluorene-centered viologen material (FBP_allyl) containing peripheral allyl moieties was coated by direct cross-linking onto graphite sheet electrodes via photoinduced thiol–ene click chemistry using pentaerythritol tetrakis­(3-mercaptopropionate) (PETMP) cross-linker under 366 nm irradiation (Scheme 1). The resulting cross-linked polymer films (FBP_allyl_X) formed homogeneous coatings on the graphite sheet electrode surface and exhibited stable capacitive behavior under dark conditions. In particular, supercapacitor measurements performed under visible light revealed a significant increase in capacitance, demonstrating the potential of thiol–ene-mediated conjugated polymers for designing photosensitive electrode interfaces in next-generation photoinduced supercapacitors.

**Scheme 1:**
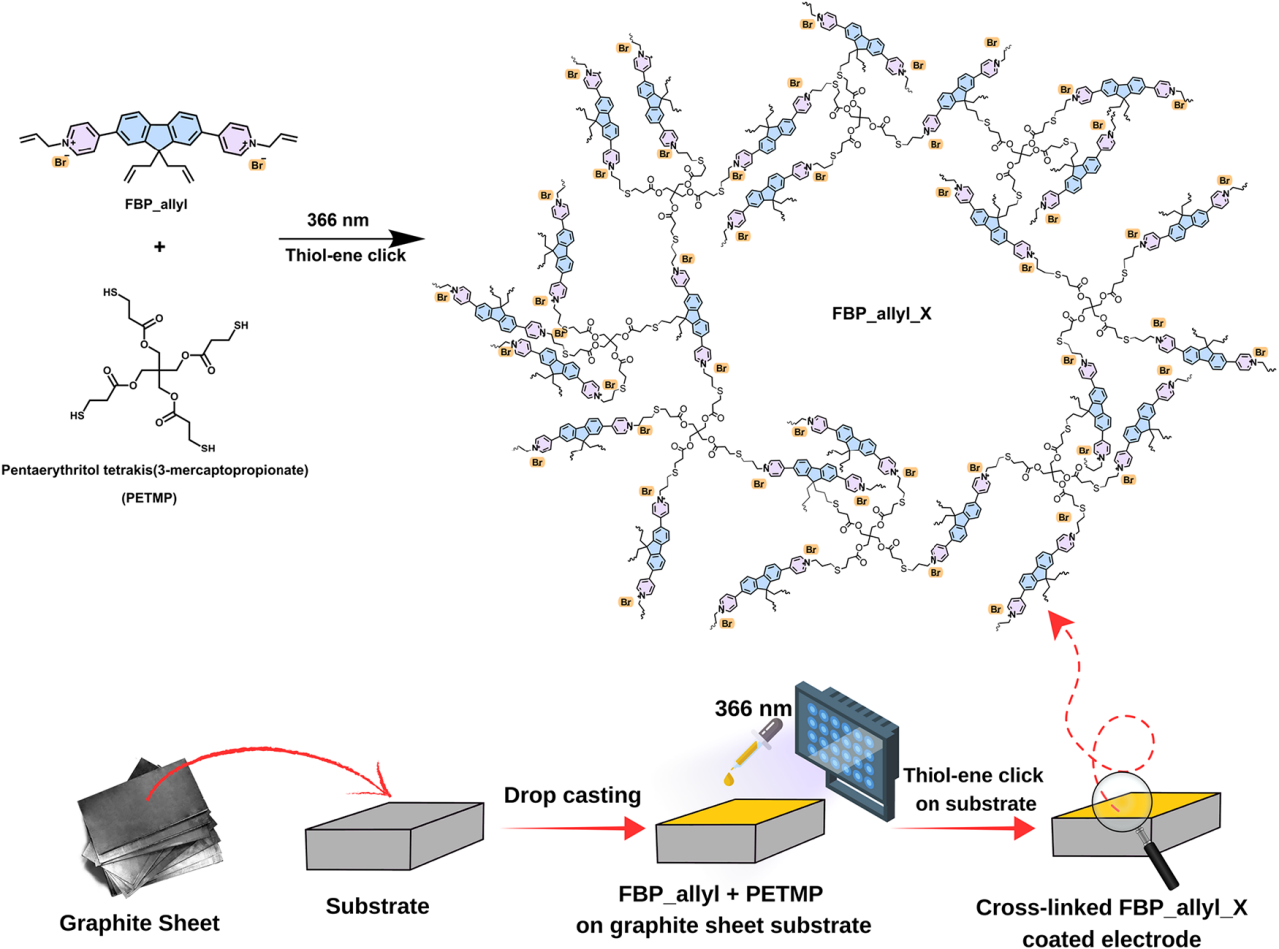
Schematic Illustration of Thiol–ene Click Cross-Linking of FBP_allyl with PETMP under UV Irradiation on a Graphite Sheet Electrode

## Results and Discussion

The synthetic route for FBP_allyl is shown in Scheme S1. In the first step, 4-pyridineboronic acid pinacol ester was coupled with 9,9-diallyl-2,7-dibromo-9H-fluorene via Suzuki coupling, followed by quaternization with allyl bromide to obtain FBP_allyl monomer. Using this monomer, cross-linked electrodes were prepared by drop casting onto graphite sheet substrates with the addition of pentaerythritol tetrakis­(3-mercaptopropionate) (PETMP) as the tetra-thiol cross-linker. The resulting films were then exposed to 366 nm UV light to trigger thiol–ene click polymerization, resulting in strong FBP_allyl_X polymer networks firmly anchored on the electrode surface (Scheme 1). Detailed experimental procedures for both monomer synthesis and electrode fabrication were provided in the Supporting Information (see SI). The molecular structure of FBP_allyl was verified by ^1^H NMR spectroscopy (Figure S1), where characteristic allyl proton signals appeared at 4.5–6.7 ppm along with aromatic resonances at 8.1–9.2 ppm, and the downfield shift of pyridinium protons confirmed quaternization. FT-IR spectra further supported the successful synthesis and cross-linking of FBP_allyl (Figure S2a,b). The FBP_allyl monomer exhibited typical C–H stretching vibrations (3085–2924 cm^–1^), allylic C–H (3001 cm^–1^), C–N^+^ (1626 cm^–1^), and C=C stretching (1600 cm^–1^). After thiol–ene reaction with PETMP, new bands at 1731 cm^–1^ (C=O), 1150–1237 cm^–1^ (C–O–C), and 1020 cm^–1^ (C–S) confirmed the formation of the cross-linked FBP_allyl_X (Figure S2a). Moreover, in the FT-IR spectra of thin films deposited on glass substrates, it was observed that FBP_allyl monomer successfully formed the cross-linked FBP_allyl_X polymer after thiol–ene reaction on the surface (Figure S2b).

The UV–vis photoluminescence spectrum of FBP_allyl monomer in methanol exhibited an intense blue-green emission with the 477 nm centered band excited from the π–π* transition at 372 nm (Figure 1a).^28^ When the UV–vis spectrum of the FBP_allyl monomer thin film was compared with the absorption band in the solution phase, a red shift of approximately 30 nm was observed with the broadened band.^29^ Furthermore, the FBP_allyl_X film showed a slight blue shift attributed to the cross-linking process. This can be explained by the reduction of intermolecular π–π interaction accompanied by the breaking of double-bonds in allyl groups during polymerization, thus interrupting the conjugation pathways (Figure 1b).^25^ The optical band gaps (Eg’) determined from the absorption onsets were 2.37 eV for the FBP_allyl film and 2.73 eV for the cross-linked FBP_allyl_X, consistent with the reduced conjugation length in the polymeric network (Figure 1b). Solid-phase photoluminescence spectra showed that the strong emission band observed in the FBP_allyl film was significantly quenched in the FBP_allyl_X film (Figure 1c). This quenching suggests possible intermolecular charge transfer interactions within the cross-linked network.^30,31^ These findings clearly demonstrate that thiol–ene cross-linking not only modifies the electronic transitions of FBP_allyl but also regulates its photophysical response, thereby producing a stable polymeric framework well-suited for efficient photoinduced charge separation and subsequent photoelectrochemical supercapacitor applications.

**Figure 1:**
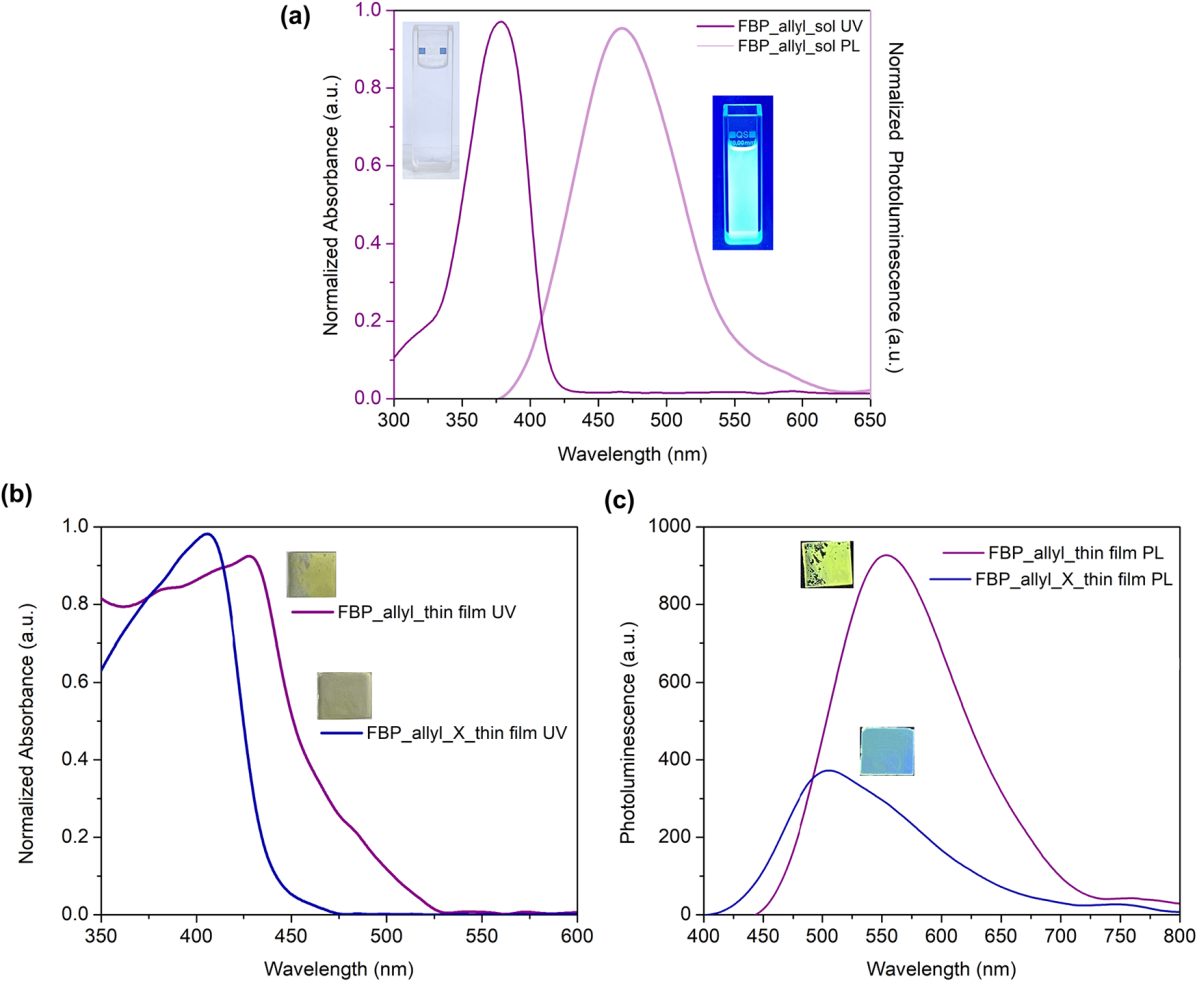
(a) UV–vis absorption and PL spectra of FBP_allyl in methanol. (b) UV–vis absorption spectra of thin films of FBP_allyl and FBP_allyl_X. (c) Photoluminescence spectra of thin films of FBP_allyl and FBP_allyl_X.

Basic electrochemical characterization was carried out using glassy carbon electrodes coated with FBP_allyl and cross-linked FBP_allyl_X films. Cyclic voltammetry (CV) analysis revealed the characteristic redox behavior of FBP_allyl and cross-linked FBP_allyl_X (Figure 2a,c). In FBP_allyl, the oxidation peak at 0.92 V is assigned to the fluorene-viologen conjugated backbone donor units, while the reduction peak at −0.71 V originates from the electron-deficient pyridinium moieties. After the cross-linking process, the oxidation peak shifted to a higher potential at 1.35 V, which can be attributed to the cleavage of allyl π-bonds in FBP_allyl. The reduction process also experienced a slight shift, indicating that the electron-accepting ability of the pyridinium units was only moderately affected by the same structural modification. Differential pulse voltammetry (DPV) provided more precise onset potentials (Figure 2b,d), which were used to estimate the frontier orbital energies. The HOMO and LUMO levels for FBP_allyl were calculated as −5.32 and −3.88 eV, respectively, resulting in an electronic band gap of 1.44 eV. FBP_allyl_X exhibited a higher electronic band gap of 1.94 eV, where the HOMO shifted to −5.73 eV and the LUMO shifted to −3.79 eV. The prolonged electronic band gap of FBP_allyl_X compared to FBP_allyl can be attributed to decreased conjugation upon allyl π-bond cleavage. Optical band gaps of 2.37 eV for FBP_allyl and 2.73 eV for FBP_allyl_X obtained from the absorption edges, compared to the electronic band gap values, show that the electrochemical and optical excitation centers are different from each other. Optical measurements indicate photoinduced π–π* transitions while electrochemical measurements reflect charge mobility and polaron formation at the electrode interface.^32^ When the results obtained from optical and electrochemical measurements are evaluated together, it is seen that FBP_allyl_X has a potential for photoelectrochemical supercapacitor applications by providing enhanced photoresponsibility and cycling durability due to its efficient light harvesting and bipolar electronic properties.

**Figure 2:**
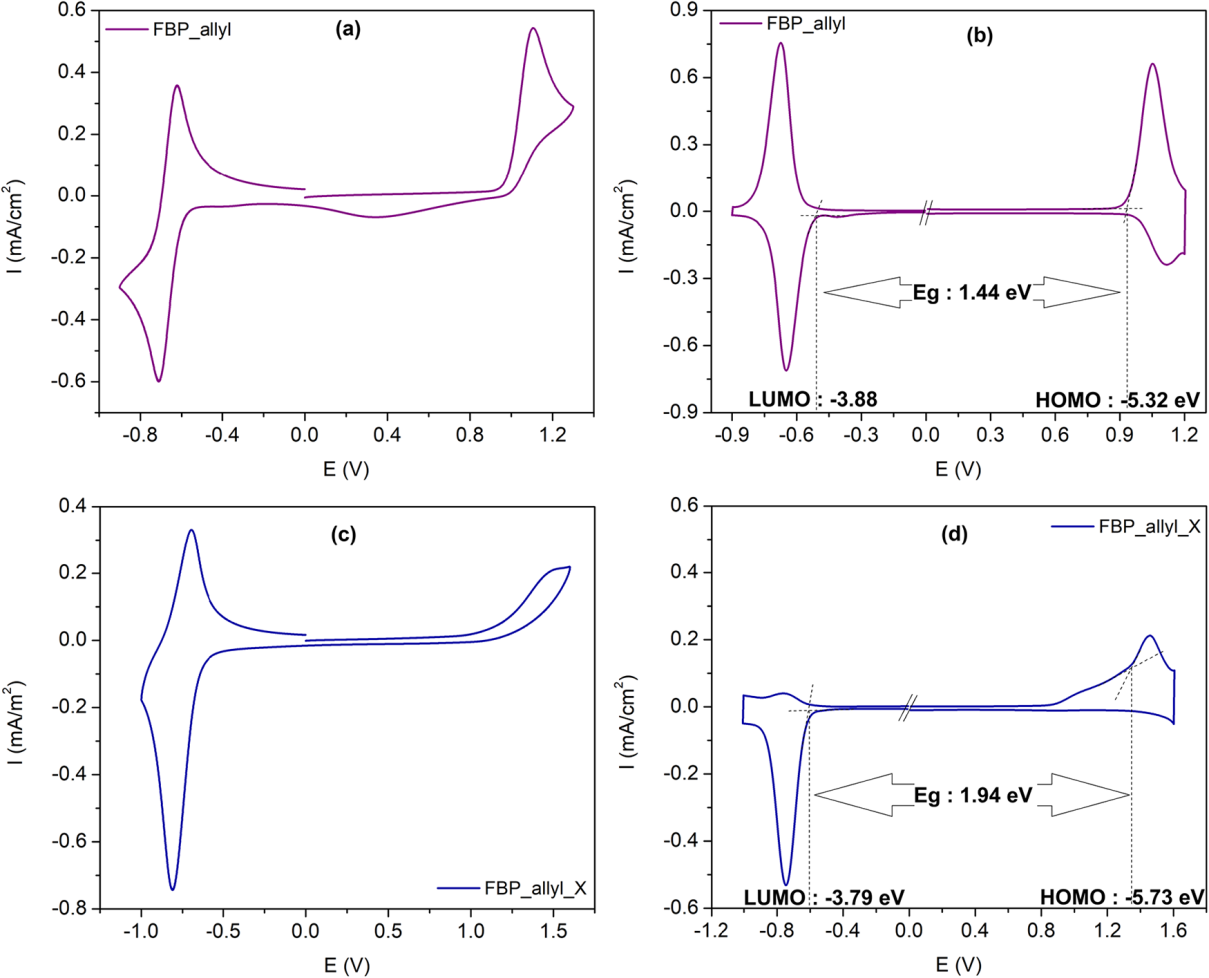
CV curves of (a) FBP_allyl, (c) FBP_allyl_X and DPV curves of (b) FBP_allyl, (d) FBP_allyl_X in a 0.1 M TBAPF_6_/ACN electrolyte solution at a scan rate of 100 mV/s.

The electronic structure and charge distribution of FBP_allyl were supported by density functional theory (DFT) calculations and compared with experimental results (Figure 3). According to the HOMO–LUMO charge distributions of FBP_allyl, at the HOMO, the charges are primarily localized on the π-conjugated fluorene-viologen backbone, consistent with its electron-donating character. At the LUMO, the charges are localized on the electron-accepting terminal pyridinium units.^33,34^ At HOMO–1, the charges are more distributed within the conjugated fluorene center, supporting efficient hole transport. Besides, at LUMO+1, partial delocalization is observed between the pyridinium moiety and neighboring π-systems. This charge distribution behavior at the HOMO and LUMO orbitals supports a good charge separation by photoexcitation. Electrochemical measurements indicate that the charge distributions are consistent with the oxidation processes associated with the fluorene central group and the reduction processes originating from the pyridinium moieties. The electronic structure and charge distribution of FBP_allyl are beneficial in reducing exciton recombination and increasing charge carrier lifetime. Furthermore, the total SCF density map shows charges delocalization throughout the conjugated network. On the other hand, Mulliken charge analysis highlighted electron-deficient pyridinium sites and electron-rich conjugated fluorene sites, consistent with the donor–acceptor nature of the molecule. Geometric optimization reveals a largely planar backbone with slight rotation at the terminal groups. Consequently, DFT calculations confirm the experimental results, indicating that the electronic structure of FBP_allyl is suitable for photoinduced charge transfer and stable charge storage in photoelectrochemical supercapacitors.

**Figure 3:**
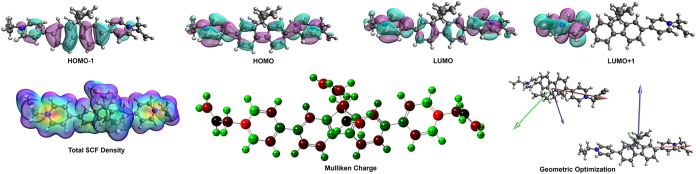
Theoretical calculations of FBP_allyl at the B3LYP/6–31G­(d,p) level.

The surface morphology and elemental composition of FBP_allyl and FBP_allyl_X deposited on graphite sheet substrates were investigated by SEM and SEM-EDX analyses (Figures 4 and S3). The FBP_allyl substrate was prepared as a reference for comparison with the cross-linked FBP_allyl_X electrode used in electrochemical applications. According to SEM images, the FBP_allyl surface exhibited relatively smooth and crystal-like domains, while the FBP_allyl_X electrode exhibited a rougher and highly porous morphology, which is characteristic of the three-dimensional polymeric network formed through cross-linking.^35–37^ SEM-EDX analyses showed C, N, and Br^–^ signals of the FBP_allyl film, while the FBP_allyl_X electrode also confirmed the increased Br^–^ content along with the S and O peaks originating from the PETMP cross-linker. Additionally, AFM measurements provided information about the surface morphology of the films on glass substrates (Figure S4). The FBP_allyl film exhibited a relatively smooth and compact surface characteristic with an average roughness (*R*_a_) of 5.5 nm. In contrast, the cross-linked FBP_allyl_X film exhibited a much rougher topography with a *R*_a_ value of 15.6 nm and height variations of up to 160 nm. This result also confirmed the formation of a three-dimensional polymer network upon formation of the cross-linked polymer.^38^ This increase in surface roughness, which supports the porous morphology of the polymer electrode in the SEM image, highlights the transformation from monomeric films to cross-linked polymer networks. Consequently, a porous and heterogeneous surface can increase the accessible surface area, thereby facilitating ion diffusion and charge storage in electrochemical applications. The thiol–ene click process not only effect the surface architecture but also ensures the robust chemical integration of the polymer network on the electrode, thus supporting FBP_allyl_X to be a stable electrode material for electrochemical energy storage systems.

**Figure 4:**
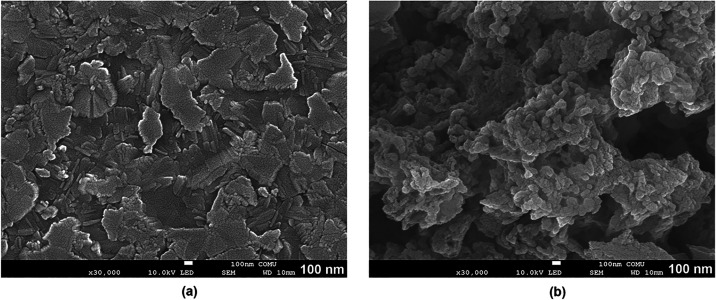
SEM images of (a) the FBP_allyl substrate and (b) the cross-linked FBP_allyl_X electrode.

The capacitive performance of the cross-linked FBP_allyl_X electrode was investigated by CV and galvanostatic charge/discharge (GCD) analyses under dark and illuminated conditions in 1 M H_2_SO_4_ electrolyte (Figures 5 and S5). The operating potential window of the FBP_allyl_X-based photoelectrochemical supercapacitor cell was also evaluated by CV and GCD measurements (Figure S6a–d). Accordingly, the photoelectrochemical cell exhibited stable and symmetric capacitive profiles without significant degradation up to 1.0 V in both conditions. Thus, considering the electrochemical stability of the cross-linked polymer electrode in this range, 0.0–1.0 V was selected as the operating window for subsequent analyses of the electrochemical cells. The CV curves taken at scan rates between 50 and 500 mV/s show quasi-rectangular shapes characteristic of the pseudocapacitive behavior of the FBP_allyl_X electrode with minimal degradation even at high scan rates. This behavior is a result of the oxidation of fluorene units and the reduction of pyridinium moieties contributing to redox-induced charge storage (Figure 5a,b).^39^ The FBP_allyl_X electrode exhibited an approximately 1.4-fold increase in current response under illumination at a scan rate of 100 mV/s compared to the dark condition, leading to a significantly expanded CV area (Figure 5c). This can be explained by the additional photoexcited charge carriers generated on the π-conjugated backbone of FBP_allyl_X promoting interfacial ion adsorption and accelerating the charge–discharge kinetics.^40^ GCD measurements further confirm the photo responsive effect of the electrode (Figure 5d–f). Under dark conditions, the GCD curve of the FBP_allyl_X electrode at 1 A/g exhibits a slight shoulder because of a faradaic contribution associated with the redox activity of the pyridinium and fluorene moieties. This situation becomes much less noticeable under illumination, and the discharge curve shifts toward a more ideal capacitive shape. This behavior can also be explained by the facilitation of charge separation between donor and acceptor groups after photoexcitation, thus improving charge transfer along the cross-linked polymer network. Furthermore, photogenerated carriers can accelerate interface redox kinetics, resulting in a more uniform triangular GCD profile. This trend is consistent with the expanded CV area and longer discharge time upon illumination. Thus, more efficient charge transfer at the electrode–electrolyte interface can increase electrochemical double-layer contribution.^41^ Additionally, it was observed that the FBP_allyl_X electrode maintained longer and more symmetric triangular profiles under illumination when the current density reached 5 A/g. Figure 5f compares the discharge time of FBP_allyl_X for the dark and light conditions at a current density of 2 A/g. The system observed an increase in discharge time under illumination, resulting in an approximately 35% improvement in charge storage. Comparing the specific capacitance values of the cell under these conditions, it was determined that the photo response behavior of the FBP_allyl_X electrode increased from 152.7 F/g in the dark to 304.1 F/g under illumination. Furthermore, the photo response behavior of the FBP_allyl_X electrode was investigated under periodic on/off illumination at zero applied bias (compared to Ag/AgCl) (Figure S7). The FBP_allyl_X electrode exhibited a highly stable and reproducible photocurrent response during abrupt transitions between light and dark states. The photocurrent amplitude reached 2.0 μA under illumination and returned to baseline in the dark. This result confirms that recombination upon material excitation is highly limited and photoinduced charge carriers are efficiently generated and separated.^42^ These results also demonstrate that thiol–ene cross-linking process not only stabilizes the polymer network on the electrode surface but also endows the system with a distinct photoenhancement effect. The increase in light-induced discharge duration, decrease in resistive losses, and broadening of capacitive response highlight the ability of FBP_allyl_X to combine stable electrochemical storage with photoactive functionality.

**Figure 5:**
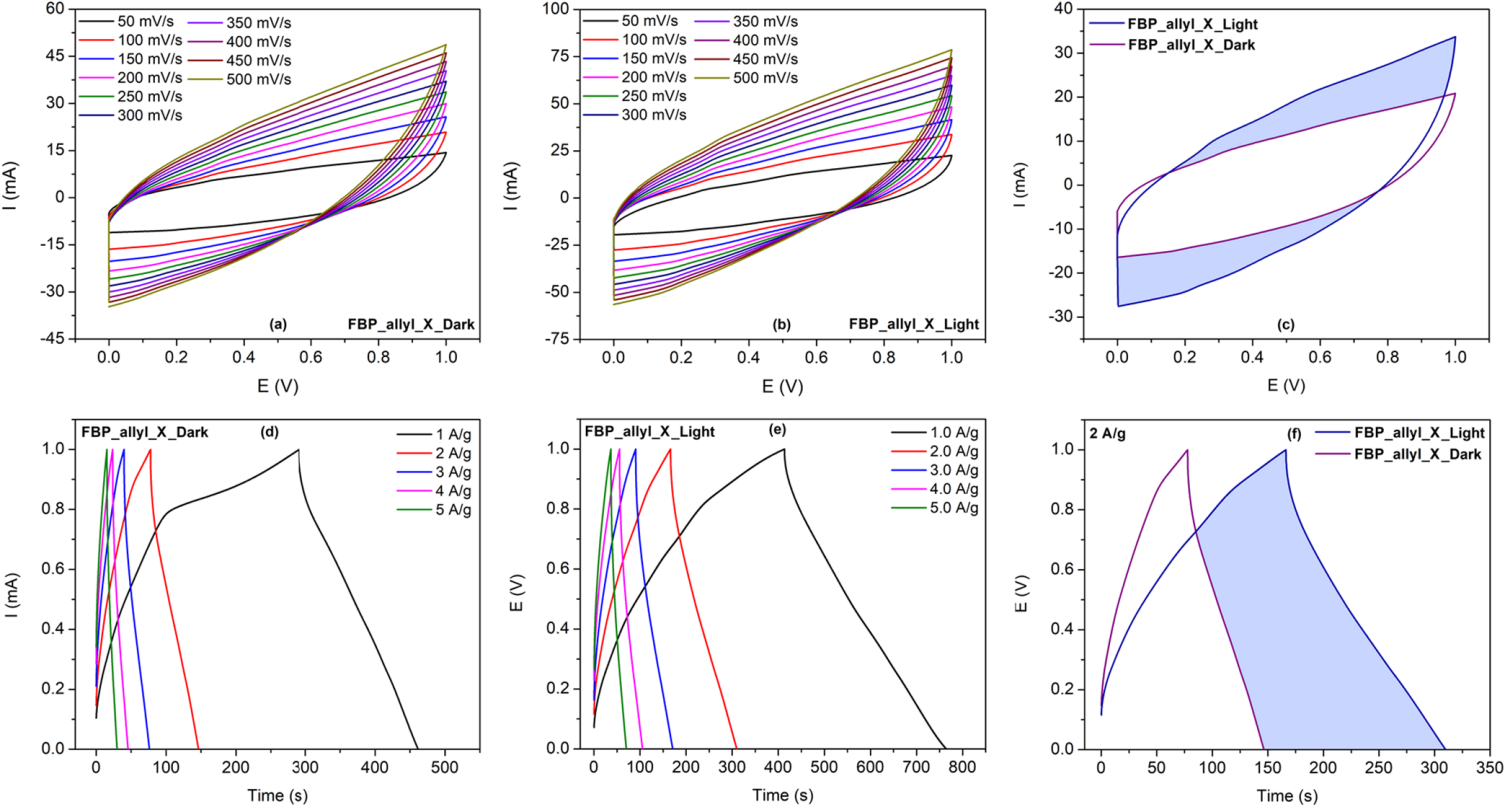
CV and GCD curves of FBP_allyl_X-based supercapacitors under dark and illuminated conditions at different scan rates and current densities: (a, b) CV curves at various scan rates in dark and under illumination, (c) CV comparison at 100 mV/s, (d, e) GCD curves at different current densities in dark and under illumination, and (f) GCD comparison at 2.0 A/g.

The charge-storage mechanism of FBP_allyl_X electrodes was further analyzed by separating capacitive and diffusion-controlled contributions using Dunn’s method (Figure 6).^43^ Under dark conditions, the capacitance was dominated by surface-controlled processes, with values exceeding 90% across all scan rates, e.g., 96.7% at 50 mV/s and remaining as high as 90.5% even at 500 mV/s (Figure 6a). This strongly indicates that pseudocapacitive reactions, likely associated with the redox-active pyridinium moieties, are the primary contributors to charge storage rather than diffusion-limited intercalation. Upon illumination, the surface-controlled contribution decreased slightly but remained the dominant mechanism, with values ranging from 94.4% at 50 mV/s to 84.2% at 500 mV/s (Figure 6b). The partial increase in surface-controlled additive illumination conditions can be explained by the relative improvement of ionic charge transport at the electrode/electrolyte interface and enhanced photoactive charge transfer.^44^ However, Dunn’s method shows that the charge storage of FBP_allyl_X is primarily dominated by fast and reversible diffusion-controlled redox reactions, consistent with the pseudocapacitive character of the pyridinium center.

**Figure 6:**
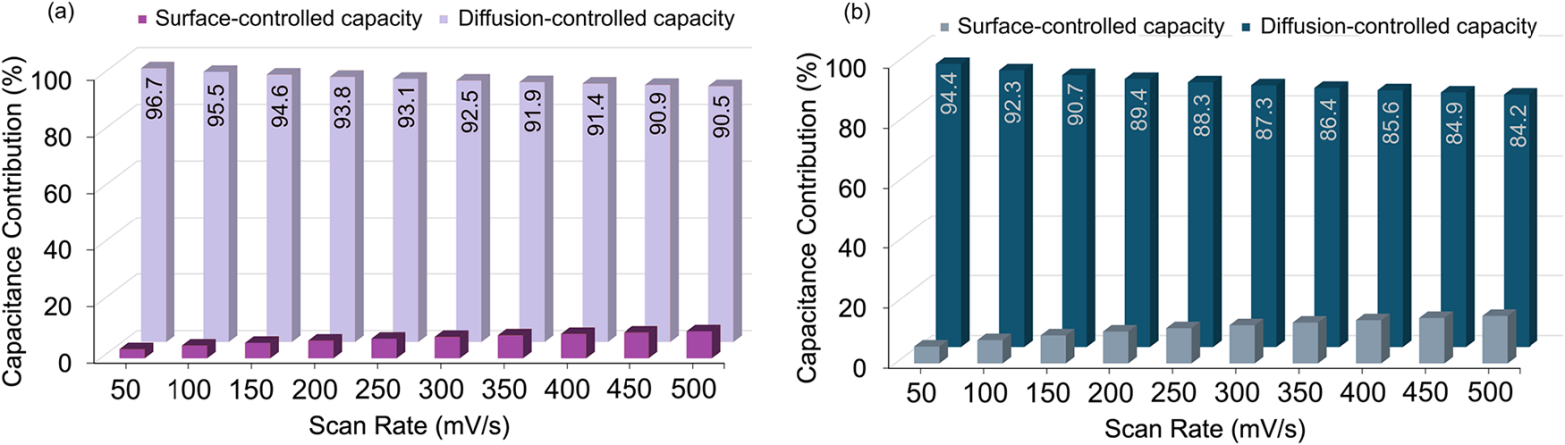
Surface-controlled and diffusion-controlled capacity (a) dark and (b) under illumination at a scan rate of 0.8 V.

Electrochemical impedance spectroscopy (EIS) revealed a significant decrease in charge transfer resistance under illumination, with a smaller semicircle (Figure 7a). This can be explained by the rapid interfacial charge transport and improved ionic conductivity under illumination. Figure 7b shows that the change in specific capacitance values with increasing current density is observed, with the specific capacitance at 1 A/g increasing from 190.2 F/g in the dark to 369.8 F/g under illumination. At a high current density of 5 A/g, the capacitance values are 76.8 and 174.4 F/g, respectively. Furthermore, the variation of specific capacitance values depending on the applied potential window and illumination conditions is investigated (Figure S8). At a current density of 3 A/g, in the dark, the specific capacitance values of the FBP_allyl_X electrode increased from 68.8 to 184.8 F/g as the potential window expanded from 0.0–0.5 to 0.0–1.2 V. Under illumination, the specific capacitance values increased from 164.2 to 413.7 F/g, an approximately 2.5-fold increase at 0.0–1.2 V potential window. Cycle stability measurements exhibited that the FBP_allyl_X electrode retained 90.6% of its capacitance in the dark and 85.7% under illumination after 10,000 cycles (Figure 7c). The relatively low stability under illumination can be attributed to photoinduced side reactions and gradual structural rearrangements of the polymer backbone.^11^ Since the EIS curve after the stability test shows continuous ion influx to and from the electrode surface after charge–discharge cycles, it is believed that there is a self-doping effect in the semiconductor polymer (Figure S9). This process increases the charge carrier density between the polymer chains, thus increasing the electrical conductivity of FBP_allyl_X based electrode. Therefore, the slightly forward shift in the EIS curve after the stability test can be attributed to increased conductivity and decreased charge transfer resistance. However, capacitive behavior also weakens slightly over longer cycles due to partial structural deterioration of the polymer chains, ion trapping, and loss of active area. The SEM image of the FBP_allyl_X electrode after the stability test exhibits that some of the polymer coating has agglomerated, with microstructural cracks and clusters forming in some areas (Figure S10). This change can be associated with rearrangement or partial degradation of the surface polymer layer as a result of prolonged potential application and repeated redox cycling. Furthermore, in this three-electrode photoelectrochemical cell configuration, the obtained energy and power density values provide predictive value for two-electrode device fabrication.^45^ Accordingly, dark conditions, the FBP_allyl_X electrode exhibited an energy density of approximately 35 Wh/kg at a maximum power density of 3676 W/kg. Under illumination, the energy density increased to 60 Wh/kg while maintaining the high-power density of 3071 W/kg. A comparative analysis is presented in Figure 7d, showing that the FBP_allyl_X electrode exhibits a very competitive performance, especially under illumination, compared to other polymer-based three-electrode supercapacitors reported in the literature.^9,46–59^ Under illumination, the specific capacitance value of FBP_allyl_X of 304.1 F/g at 2.0 A/g is higher than many similar systems. When the results under light conditions are evaluated together with the value of 152.7 F/g at 2 A/g under dark conditions, the FBP_allyl_X electrode is promising in terms of both photo response and overall energy storage efficiency.

**Figure 7:**
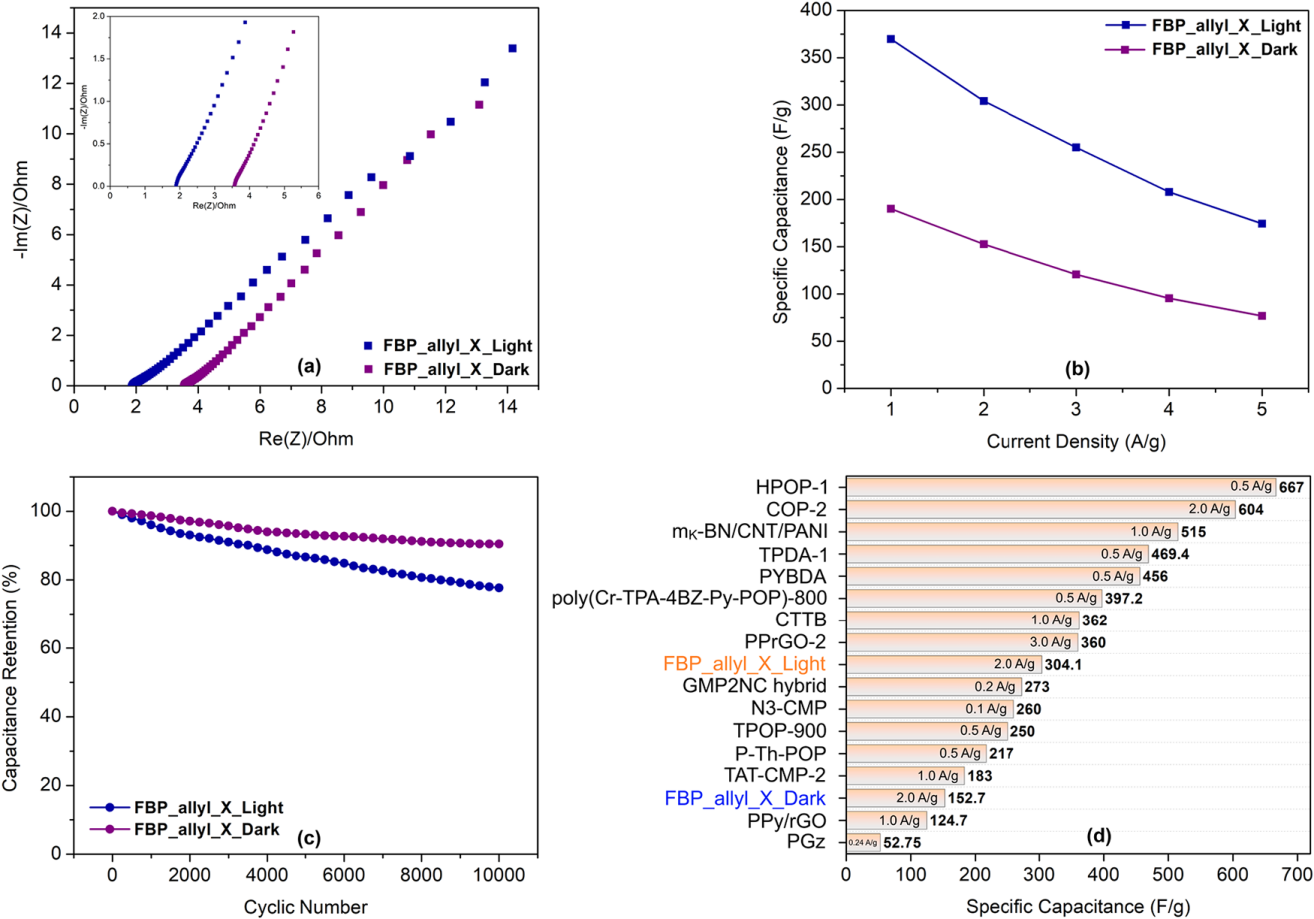
(a) Nyquist plots, (b) specific capacitance at different current densities, (c) cycling stability at 2 A/g, and (d) Comparison of the specific capacitance of the FBP_allyl_X electrode with other reported polymer-based three-electrode supercapacitor cells.^9,46–59^

Consequently, photoelectrochemical supercapacitors remain a relatively new class of devices that aim to combine photon harvesting with charge storage on a single platform.^60^ Among the limited reports on photoelectrochemical supercapacitors, Safshekan et al. reported a BiVO_4_/PbO_*x*_ heterostructure photocapacitor, combining the visible-light absorption of BiVO_4_ with the pseudocapacitive redox activity of PbO_*x*_, which delivered a specific capacitance of 6 mF/cm^2^ along with a high open-circuit potential of 1.5 V vs RHE and stable cycling behavior.^15^ In another example, Zhu et al. introduced a Fe_2_O_3_@Ni­(OH)_2_ core–shell nanorod array, in which Fe_2_O_3_ harvested light to generate electron–hole pairs while Ni­(OH)_2_ stored photogenerated holes, yielding a capacitance of 20.6 mF/cm^2^ at 0.1 mA/cm^2^, about 4.5 times higher than that of BiVO_4_/PbO_*x*_ electrodes.^13^ An et al. further advanced this concept by fabricating a nanoporous Cu@Cu_2_O (NPC@Cu_2_O) hybrid array electrode, achieving a high capacitance of 782 F/g at 1 A/g under illumination, which represented a 37.9% enhancement over the dark state due to photoinduced hole accumulation and proton insertion in Cu_2_O facets.^14^ Although most reported photoelectrochemical supercapacitors have relied on inorganic semiconductors such as Fe_2_O_3_, Cu_2_O, or BiVO_4_ to couple light harvesting with charge storage, only a handful of studies have explored organic frameworks in this context. Podjaski et al. demonstrated that cyanamide-functionalized poly heptazine imide (NCN-PHI), a 2D graphitic carbon nitride, could simultaneously act as a light absorber and pseudocapacitive anode, storing photogenerated charges for hours.^61^ In another example, Lee et al. reported flexible capacitors based on conjugated polymers (e.g., P3HT), where illumination triggered up to a 3-fold enhancement in capacitance, highlighting the feasibility of fully organic light-responsive storage.^62^ Recently, the fabrication of polymer-based electrodes that are both photosensitive and resistant to electrochemical cycling has attracted considerable interest. In this study, FBP_allyl_X, which combines thiol–ene cross-linking with photoactivity, may be an effective candidate for durable, photosensitive polymer-based supercapacitors with acceptable capacitive performance.

## Conclusions

In this study, FBP_allyl_X electrodes were fabricated on the surface of conductive graphite sheet via a thiol–ene click reaction. In a three-electrode photoelectrochemical cell, the FBP_allyl_X electrode exhibited impressive capacitive performance. At a current density of 2 A/g, the electrode provided a specific capacitance of 152.7 F/g in the dark, while this value increased to 304.1 F/g under illumination. Energy-power analysis based on the three-electrode configuration revealed a significant increase in energy density from 35 to 60 Wh/kg, while providing high power densities of 3676 and 3071 W/kg in the dark and under illumination, respectively. The cell exhibited long-term stability, retaining more than 85% of its capacitance under illumination after 10,000 cycles. The results confirmed that the FBP_allyl_X electrode exhibits efficient charge separation, fast ion transport, and durable electrochemical performance due to the strong synergy between the favorable donor–acceptor molecular design and the thiol–ene cross-linking. The energy storage performance of FBP_allyl_X via a photoelectrochemical cell demonstrates the potential of this material as a new class of polymer-based, photoassisted supercapacitors that bridge the gap between conventional capacitors and novel hybrid energy storage devices.

## Supplementary Material



## Notes

Preparation of FBP_allyl and FBP_allyl_X graphite sheet electrodes, synthetic route for FBP_allyl, ^1^H NMR spectrum, FT-IR spectra in powder and film states, SEM-EDX analysis, AFM characterization, schematic illustration of photoelectrochemical supercapacitor assembly, CV and GCD curves under dark and illuminated conditions, and photoresponse of FBP_allyl_X electrode under periodic light on/off cycling (PDF)

## Abbreviations

SCs: supercapacitors
EDLC: electric double-layer capacitors
PETMP: pentaerythritol tetrakis­(3-mercaptopropionate)
Eg’: optical band gaps
CV: cyclic voltammetry
DPV: differential pulse voltammetry
DFT: density functional theory
Ra: average roughness
GCD: galvanostatic charge/discharge
EIS: electrochemical impedance spectroscopy

## References

[1] Njema G. G., Ouma R. B. O., Kibet J. K. (2024). A review on the recent advances in
battery development and energy storage technologies. J. Renew. Energy.

[2] Gan Z., Yin J., Xu X., Cheng Y., Yu T. (2022). Nanostructure and advanced
energy storage: elaborate material designs lead to high-rate pseudocapacitive
ion storage. ACS Nano.

[3] Liu L., Zhang X., Liu Y., Gong X. (2025). Electrochemical energy
storage devices batteries, supercapacitors, and battery–supercapacitor
hybrid devices. ACS Appl. Electron. Mater..

[4] Zhu Z., Jiang T., Ali M., Meng Y., Jin Y., Cui Y., Chen W. (2022). Rechargeable
batteries for grid scale energy storage. Chem.
Rev..

[5] Dutta A., Mitra S., Basak M., Banerjee T. (2023). A comprehensive
review
on batteries and supercapacitors: Development and challenges since
their inception. Energy Storage.

[6] Panchu S. J., Raju K., Swart H. C. (2024). Emerging two–dimensional intercalation
pseudocapacitive electrodes for supercapacitors. ChemElectroChem.

[7] Tan J., Li Z., Ye M., Shen J. (2022). Nanoconfined space: Revisiting the
charge storage mechanism of electric double layer capacitors. ACS Appl. Mater. Interfaces.

[8] Çatoğlu F., Altınışık S., Koyuncu S. (2024). Comparative Study of
Electrochromic Supercapacitor Electrodes Based on PEDOT: PSS/ITO Fabricated
via Spray and Electrospray Methods. ACS omega.

[9] Ermis S., Altinisik S., Catoglu F., Yagci Y., Sari E., Jockusch S., Koyuncu S., Kaya K. (2025). From Plant Oils to
High-Performance Supercapacitor Electrode: Poly (guaiazulene) via
Photopolymerization. Adv. Electron. Mater..

[10] Chatterjee D. P., Nandi A. K. (2021). A review on the
recent advances in hybrid supercapacitors. J.
Mater. Chem. A.

[11] K N., Rout C. S. (2021). Photo-powered integrated supercapacitors: a review
on recent developments, challenges and future perspectives. J. Mater. Chem. A.

[12] Wang L., Wen L., Tong Y., Wang S., Hou X., An X., Dou S. X., Liang J. (2021). Photo-rechargeable batteries and
supercapacitors: Critical roles of carbon-based functional materials. Carbon Energy.

[13] Zhu K., Zhu G., Wang J., Zhu J., Sun G., Zhang Y., Li P., Zhu Y., Luo W., Zou Z., Huang W. (2018). Direct storage
of holes in ultrathin Ni (OH) 2 on Fe2O3 photoelectrodes for integrated
solar charging battery-type supercapacitors. J. Mater. Chem. A.

[14] An C., Wang Z., Xi W., Wang K., Liu X., Ding Y. (2019). Nanoporous Cu@ Cu 2 O hybrid arrays enable photo-assisted supercapacitor
with enhanced capacities. J. Mater. Chem. A.

[15] Safshekan S., Herraiz-Cardona I., Cardenas-Morcoso D., Ojani R., Haro M., Gimenez S. (2017). Solar Energy
Storage by a Heterostructured BiVO4–PbO
x Photocapacitive Device. ACS Energy Lett..

[16] Zhang P., Wang T., Chang X., Gong J. (2016). Effective charge carrier
utilization in photocatalytic conversions. Acc.
Chem. Res..

[17] Golriz M., Hosseini Sharifi Kalahroudi H., Moghari S., Dawi E. A., Khonakdar H. A. (2025). The Role
of Phase-Transition and Electrically Active
Polymers in Energy Storage and Harvesting Applications: A Review. Polym. Eng. Sci..

[18] Li J., Jing X., Li Q., Li S., Gao X., Feng X., Wang B. (2020). Bulk COFs and COF nanosheets
for
electrochemical energy storage and conversion. Chem. Soc. Rev..

[19] Ejaz M., Mohamed M. G., Chang W. C., Kuo S. W. (2024). Synthesis and design
of hypercrosslinked porous organic frameworks containing tetraphenylpyrazine
unit for high-performance supercapacitor. J.
Polym. Sci..

[20] Lowe A. B. (2010). Thiol-ene
“click” reactions and recent applications in polymer
and materials synthesis. Polym. Chem..

[21] Degirmenci A., Sanyal R., Sanyal A. (2024). Metal-free click-chemistry: a powerful
tool for fabricating hydrogels for biomedical applications. Bioconjugate Chem..

[22] Davis A. R., Carter K. R. (2014). Surface grafting
of vinyl-functionalized poly (fluorene)
s via thiol–ene click chemistry. Langmuir.

[23] Davis A. R., Maegerlein J. A., Carter K. R. (2011). Electroluminescent networks via photo
“click” chemistry. J. Am. Chem.
Soc..

[24] Yang K., Huang X., Zhu M., Xie L., Tanaka T., Jiang P. (2014). Combining RAFT polymerization and thiol–ene click reaction
for core–shell structured polymer@ BaTiO3 nanodielectrics with
high dielectric constant, low dielectric loss, and high energy storage
capability. ACS Appl. Mater. Interfaces.

[25] Özdemir M., Altınışık S., Ömeroğlu İ., Köksoy B., Durmuş M., Yalçın B., Koyuncu S. (2023). Direct Photopatterning
of BODIPY-Based
Small Molecules via Thiol-ene Click Chemistry. ChemNanoMat.

[26] Harant A. W., Khire V. S., Thibodaux M. S., Bowman C. N. (2006). Thiol– Ene
Photopolymer Grafts on Functionalized Glass and Silicon Surfaces. Macromolecules.

[27] Uluçay S., Ha N. G., Kortun A., Altınışık S., Pıravadılı S., Kwon J. H., Moon H. C., Koyuncu S. (2025). Electrochromic supercapacitor
electrodes based on viologen-derived
cross-linked thin films. Org. Electron..

[28] Ni M., An X., Bai L., Wang K., Cai J., Wang S., He L., Xu M., Liu H., Lin J. (2022). Intrinsically
Stretchable and Stable Ultra-Deep-Blue Fluorene-Based Polymer with
a High Emission Efficiency of≈ 90% for Polymer Light-Emitting
Devices with a CIEy= 0.06. Adv. Funct. Mater..

[29] Martin K. L., Krishnamurthy A., Strahan J., Young E. R., Carter K. R. (2019). Excited
state characterization of carborane-containing poly (dihexyl fluorene)
s. J. Phys. Chem. A.

[30] Xiao Y., Lei X., Zhang Z., Chen S., Xiong G., Ma X., Zhang Q. (2024). Carbazole-based
polyimide membranes with hydrogen-bonding interactions
for gas separation. Macromolecules.

[31] Healy A. T., Boudouris B. W., Frisbie C. D., Hillmyer M. A., Blank D. A. (2013). Intramolecular
exciton diffusion in poly (3-hexylthiophene). J. Phys. Chem. Lett..

[32] Aderne R. E., Borges B. G. A., Ávila H. C., von Kieseritzky F., Hellberg J., Koehler M., Cremona M., Roman L. S., Araujo C. M., Rocco M. L. M., Marchiori C. F. N. (2022). On the
energy gap determination of organic optoelectronic materials: the
case of porphyrin derivatives. Mater. Adv..

[33] Altınışık S., Yıldız G., Hatay Patır Im., Koyuncu S. (2024). Boosting photocatalytic
metal-free hydrogen production of viologen-based covalent organic
frameworks. ACS Appl. Eng. Mater..

[34] Jegorovė A., Daškevičienė M., Kantminienė K., Jankauskas V., Čepas R. J., Gruodis A., Getautis V., Genevičius K. (2024). New fluorene-based
bipolar charge transporting materials. RSC Adv..

[35] Uyumaz F., Nurgaziyeva E., Kalybekkyzy S., Kahraman M. V. (2024). Thiol-Ene Photo
Crosslinked PUA-PUMA-Based Flexible Gel Polymer Electrolyte for Lithium-Ion
Batteries. Macromol. Mater. Eng..

[36] Wessely I. D., Matt Y., An Q., Bräse S., Tsotsalas M. (2021). Dynamic porous organic polymers with tuneable crosslinking
degree and porosity. RSC Adv..

[37] Zheng X., Luo J., Lv W., Wang D. W., Yang Q. H. (2015). Two-dimensional
porous carbon: synthesis and ion-transport properties. Adv. Mater..

[38] Hobiger V., Zahoranova A., Baudis S., Liska R., Krajnc P. (2021). Thiol–Ene
Cross-linking of Poly (ethylene glycol) within high internal phase
emulsions: Degradable hydrophilic PolyHIPEs for controlled drug release. Macromolecules.

[39] Li Y., Zhang M., Lu H., Cai X., Jiao Z., Li S., Song W. (2024). Boosting High-Performance
Aqueous Zinc-Ion Hybrid Capacitors
via Organic Redox Species on Laser-Induced Graphene Network. Adv. Funct. Mater..

[40] Zhou T., Wang N., Gao Y., Li X. (2025). Cation−π
interactions in polymer science: from fundamental insights to material
applications. Polym. Chem..

[41] Ge X., Fu C., Chan S. H. (2011). Double
layer capacitance of anode/solid-electrolyte
interfaces. Phys. Chem. Chem. Phys..

[42] Wang Y., Zheng H., Xiao J., Liu Y., Liu Q., Ma X., Hu J., Zou D., Hou S. (2024). Intensity-Modulated
Photocurrent and Photovoltage Spectroscopy for Characterizing Charge
Dynamics in Solar Cells. Adv. Energy Mater..

[43] Wang J., Polleux J., Lim J., Dunn B. (2007). Pseudocapacitive contributions
to electrochemical energy storage in TiO2 (anatase) nanoparticles. J. Phys. Chem. C.

[44] Li T., Zhang Y., Li L., Ren K., Yao J., Zhang W., Zhang Y., Liu H., Li J. (2025). Advances on
Emerging Integrated Photocapacitors: Strategies, Design, and Challenges. Small.

[45] Lv J., Xie J., Mohamed A. G. A., Zhang X., Wang Y. (2022). Photoelectrochemical
energy storage materials: design principles and functional devices
towards direct solar to electrochemical energy storage. Chem. Soc. Rev..

[46] Xu P., Ouyang S., Bai Q., Ma Q., Zhu Y. (2024). A hexaazatriphenylene-based
porous organic polymer for high performance supercapacitor. J. Polym. Sci..

[47] Ambrose B., Nasrin K., Arunkumar M., Kannan A., Sathish M., Kathiresan M. (2023). Viologen-based
covalent organic polymers: Variation
of morphology and evaluation of their ultra-long cycle supercapacitor
performance. J. Energy Storage.

[48] Maity C. K., Sahoo S., Verma K., Behera A. K., Nayak G. C. (2020). Facile
functionalization of boron nitride (BN) for the development of high-performance
asymmetric supercapacitors. New J. Chem..

[49] Bhanja P., Das S. K., Bhunia K., Pradhan D., Hayashi T., Hijikata Y., Irle S., Bhaumik A. (2018). A new porous polymer
for highly efficient capacitive energy storage. ACS Sustainable Chem. Eng..

[50] Bandyopadhyay S., Singh C., Jash P., Hussain M. W., Paul A., Patra A. (2018). Redox-active, pyrene-based
pristine porous organic polymers for efficient
energy storage with exceptional cyclic stability. ChemComm.

[51] Mohamed M. G., Su B. X., Kuo S. W. (2024). Robust nitrogen-doped microporous
carbon via crown ether-functionalized benzoxazine-linked porous organic
polymers for enhanced CO2 adsorption and supercapacitor applications. ACS Appl. Mater. Interfaces.

[52] Özdemir M., Uluçay S., Sevimli E., Altınışık S., Köksoy B., Yalçın B., Koyuncu S. (2025). Light-Induced Performance
Enhancement of Supercapacitors through Thiol-Ene Click Surface Functionalization
of Thienothiophene-BODIPY Porous Polymers. ACS
Appl. Energy Mater..

[53] Sajjad M., Tao R., Kang K., Luo S., Qiu L. (2021). Phosphine-based porous
organic polymer/rGO aerogel composites for high-performance asymmetric
supercapacitor. ACS Appl. Energy Mater..

[54] Yuan K., Hu T., Xu Y., Graf R., Shi L., Forster M., Pichler T., Riedl T., Chen Y., Scherf U. (2017). Nitrogen-doped
porous carbon/graphene nanosheets derived from two-dimensional conjugated
microporous polymer sandwiches with promising capacitive performance. Mater. Chem. Front..

[55] Lee J. S. M., Wu T. H., Alston B. M., Briggs M. E., Hasell T., Hu C. C., Cooper A. I. (2016). Porosity-engineered
carbons for supercapacitive
energy storage using conjugated microporous polymer precursors. J. Mater. Chem. A.

[56] Yang M., Long X., Li H., Chen H., Liu P. (2019). Porous organic-polymer-derived
nitrogen-doped porous carbon nanoparticles for efficient oxygen reduction
electrocatalysis and supercapacitors. ACS Sustainable
Chem. Eng..

[57] Mohamed M. G., Chaganti S. V., Li M. S., Samy M. M., Sharma S. U., Lee J. T., Elsayed M. H., Chou H. H., Kuo S. W. (2022). Ultrastable
porous organic polymers containing thianthrene and pyrene units as
organic electrode materials for supercapacitors. ACS Appl. Energy Mater..

[58] Li X. C., Zhang Y., Wang C. Y., Wan Y., Lai W. Y., Pang H., Huang W. (2017). Redox-active triazatruxene-based
conjugated microporous polymers for high-performance supercapacitors. Chem. Sci..

[59] Lau S. C., Lim H. N., Ravoof T. B. S. A., Yaacob M. H., Grant D. M., MacKenzie R. C. I., Harrison I., Huang N. M. (2017). A three-electrode
integrated photo-supercapacitor utilizing graphene-based intermediate
bifunctional electrode. Electrochim. Acta.

[60] Flores-Diaz N., De Rossi F., Das A., Deepa M., Brunetti F., Freitag M. (2023). Progress of photocapacitors. Chem. Rev..

[61] Podjaski F., Kröger J., Lotsch B. V. (2018). Toward an aqueous solar battery:
direct electrochemical storage of solar energy in carbon nitrides. Adv. Mater..

[62] Lee H., Kim J., Kim H., Kim Y. (2016). Strong photo-amplification effects
in flexible organic capacitors with small molecular solid-state electrolyte
layers sandwiched between photo-sensitive conjugated polymer nanolayers. Sci. Rep..

