# Supplementary material for: Light-Induced Enhancement of Cross-Linked Viologen-Fluorene Polymer Electrode for High-Performance Supercapacitors
Source: ACS Omega. 2025 Dec 19;11(1):1826–35. doi: 10.1021/acsomega.5c09977 (PMC12809359; doi:10.1021/acsomega.5c09977)
Supplement: Supplementary file 1 [file ao5c09977_si_001.pdf]

Supporting Information

Light-Induced Enhancement of Crosslinked Viologen-Fluorene Polymer Electrode for High-Performance Supercapacitors

*Sinem Altınışık**

Table of Contents

**Preparation of FBP_allyl and FBP_allyl_X graphite sheet electrodes.**	**3**
Scheme S1. Synthetic route for the preparation of FBP_allyl.	3
**Materials Characterizations.**	**4**
Figure S1. ^{1}H-NMR spectrum of FBP_allyl (solvent: DMSO-d_6).	6
Figure S2. FT-IR spectra of (a) FBP_allyl and FBP_allyl_X in powder form and (b) FBP_allyl and FBP_allyl_X deposited on glass substrates.	7
Figure S3. SEM-EDX patterns of FBP_allyl and FBP_allyl_X electrodes.	8
Figure S4. AFM images of FBP_allyl and FBP_allyl_X films on glass substrates.	9
**Three-Electrode Photoelectrochemical Supercapacitor Assembly.**	**10**
Figure S5. Schematic illustration of photoelectrochemical supercapacitor (a) dark and (b) under illumination.	10
Figure S6. CV (a, b) and GCD (c, d) curves of the device under dark and illuminated conditions at different potential windows.	12
Figure S7. Photo-response of the FBP_allyl_X electrode under periodic on/off light illumination at zero applied bias (Ag/AgCl).	13
Figure S8. (a) Specific capacitance of FBP_allyl_X in the dark and (b) under illumination at different potential windows.	14
Figure S9. Nyquist plots of FBP_allyl_X electrode before and after the cyclic stability test.	15
Figure S10. SEM images of FBP_allyl_X electrode after the cyclic stability test.	16
**References**	**17**

Preparation of FBP_allyl and FBP_allyl_X graphite sheet electrodes.

The synthesis of FBP_allyl was accomplished by a slightly modified two-step procedure.[1,2] In the first step, 9,9-diallyl-2,7-dibromo-9H-fluorene (1.2 g, 2.97 mmol) and 4-pyridineboronic acid pinacol ester (1.34 g, 6.53 mmol) were dissolved in a mixed solvent of toluene/DMF (2:1) together with aqueous 3.0 M K_2CO_3 (1.23 g, 8.90 mmol) in a Schlenk flask. After purging with argon for 15 min, $Pd(PPh_3)_4$ was introduced as the catalyst, and the reaction mixture was stirred at 110 °C for 8 h under an inert atmosphere. Following completion, the mixture was cooled to room temperature and poured into ethanol, affording a precipitate that was collected by filtration, washed with ethanol, and dried under vacuum at 60 °C to give 4,4'-(9,9-diallyl-9H-fluorene-2,7-diyl)dipyridine. In the subsequent step, this intermediate (0.8 g, 2.00 mmol) was dissolved in acetonitrile and treated with allyl bromide (0.50 mL, 5.79 mmol). The sealed flask was heated at 80 °C for 6 h, after which the resulting solid precipitate was isolated by filtration and recrystallized from ethanol. The final product, 4,4'-(9,9-diallyl-9H-fluorene-2,7-diyl)bis(1-allylpyridinium) dibromide (FBP_allyl), was obtained as a yellowish-brown powder after drying under vacuum at 80 °C overnight (yield: 86%) (Scheme S1).

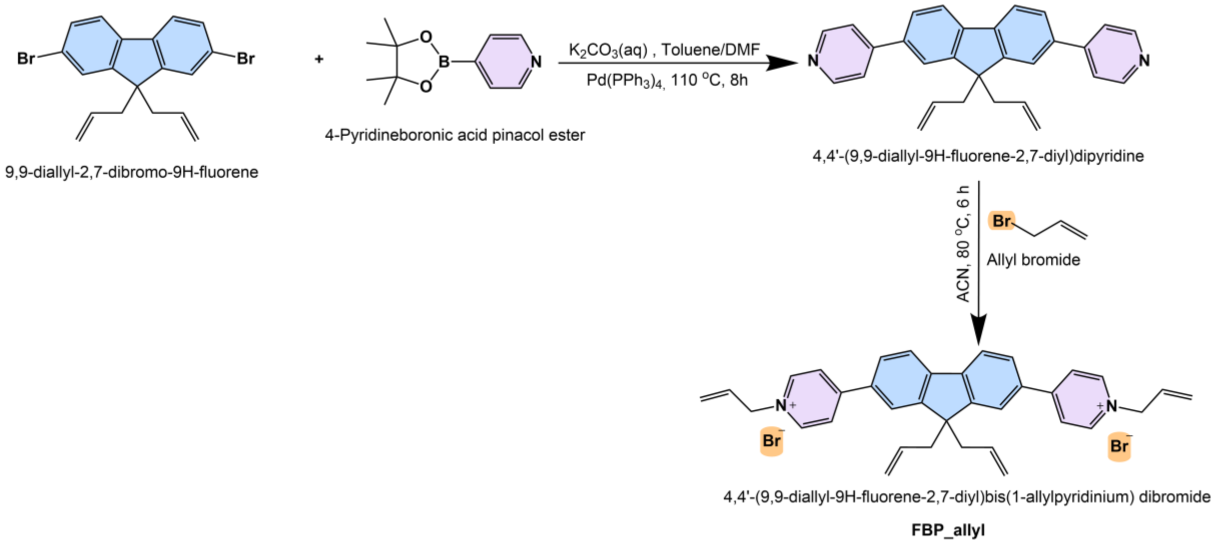

Scheme S1. Synthetic route for the preparation of FBP_allyl.

Graphite sheet electrodes (1.0 x 3.0 cm) were masked to define a geometric active area (typically 1.0 x 1.0 cm^2), sequentially cleaned by ultrasonication in acetone, isopropanol, and deionized water, and dried under a nitrogen stream. FBP_allyl was dissolved in methanol at a concentration of 1.5 mg/mL, filtered through a 0.22 µm PTFE membrane, and stored in amber vials to prevent photodegradation. As a cross-linker, pentaerythritol tetrakis(3-mercaptopropionate) (0.5 mg per mL of FBP_allyl solution) was added with gentle stirring until a homogeneous solution was obtained. The resulting solution was drop-cast onto the preheated graphite substrates (50 ± 2 °C) at a loading of 1 mL/cm^2. Each electrode contains approximately 1.5 mg of active material (FBP_allyl) and 0.5 mg of cross-linker, resulting in a total polymer layer of about 2 mg per cm^2. To control spreading and evaporation, the electrodes were covered with a clean glass Petri dish and left for 2 min to allow partial solvent removal. Subsequently, the electrodes were irradiated with a 366 nm UV source (5-15 mW/cm, lamp-substrate distance 8 cm) for 15 min at 50 °C. This photo-activated thiol-ene cross-linking process facilitated strong adhesion of FBP_allyl to the graphite surface, yielding cross-linked FBP_allyl_X polymer films on the electrodes.

Materials Characterizations.

The structural and electronic properties of the compounds were examined using a combination of spectroscopic, electrochemical, and microscopic methods. Fourier-transform infrared spectroscopy (FT-IR, Agilent Cary 630) and proton nuclear magnetic resonance (^1H NMR, JEOL ECX-400, 400 MHz, in DMSO-d_6) were employed to confirm molecular structures and chemical environments. Optical absorption spectra were acquired on an Analytic Jena Speedcord S-600 diode-array spectrophotometer equipped with a deuterium lamp over the 250-1100 nm range. Optical band gaps (Eg) of FBP_allyl and FBP_allyl_X were estimated from the absorption onset (λ_{onset}) values using the relation Eg = 1241/λ_{onset}.[3] Photoluminescence (PL) measurements were performed on a PTI QM1 spectrofluorometer. Electrochemical measurements were carried out on a BioLogic SP-50e workstation under an argon atmosphere. Cyclic voltammetry (CV) and differential pulse voltammetry (DPV) experiments employed a three-electrode configuration consisting of a glassy carbon working electrode, a platinum wire counter electrode, and an Ag/AgCl reference electrode, with 0.1 M

tetrabutylammonium hexafluorophosphate ($TBAPF_6$) in acetonitrile as the supporting electrolyte. HOMO and LUMO levels were calculated from the oxidation and reduction onset potentials referenced to the ferrocene/ferrocenium (Fc/Fc^+) couple, according to the equations: E_{HOMO}= -e ($E_{ox\text{-}ons}$ - E_{Fc}) + (-4.8 eV) and E_{LUMO}= -e ($E_{red\text{-}ons}$ - E_{Fc}) + (-4.8 eV).[4] Density functional theory (DFT) calculations were carried out using Gaussian16 with the B3LYP functional and the 6-31G(d,p) basis set, and optimized geometries were visualized using IQMol v2.14.0.[5-7] Surface morphologies were studied by scanning electron microscopy (SEM, JEOL JSM-7100F), while atomic force microscopy (AFM) measurements were performed in noncontact mode (wave mode) at ambient conditions, typically over 10 μm × 10 μm scan areas. The elemental composition and distribution of FBP_allyl and FBP_allyl_X were further analyzed using energy-dispersive X-ray spectroscopy (EDX) integrated with the SEM instrument.

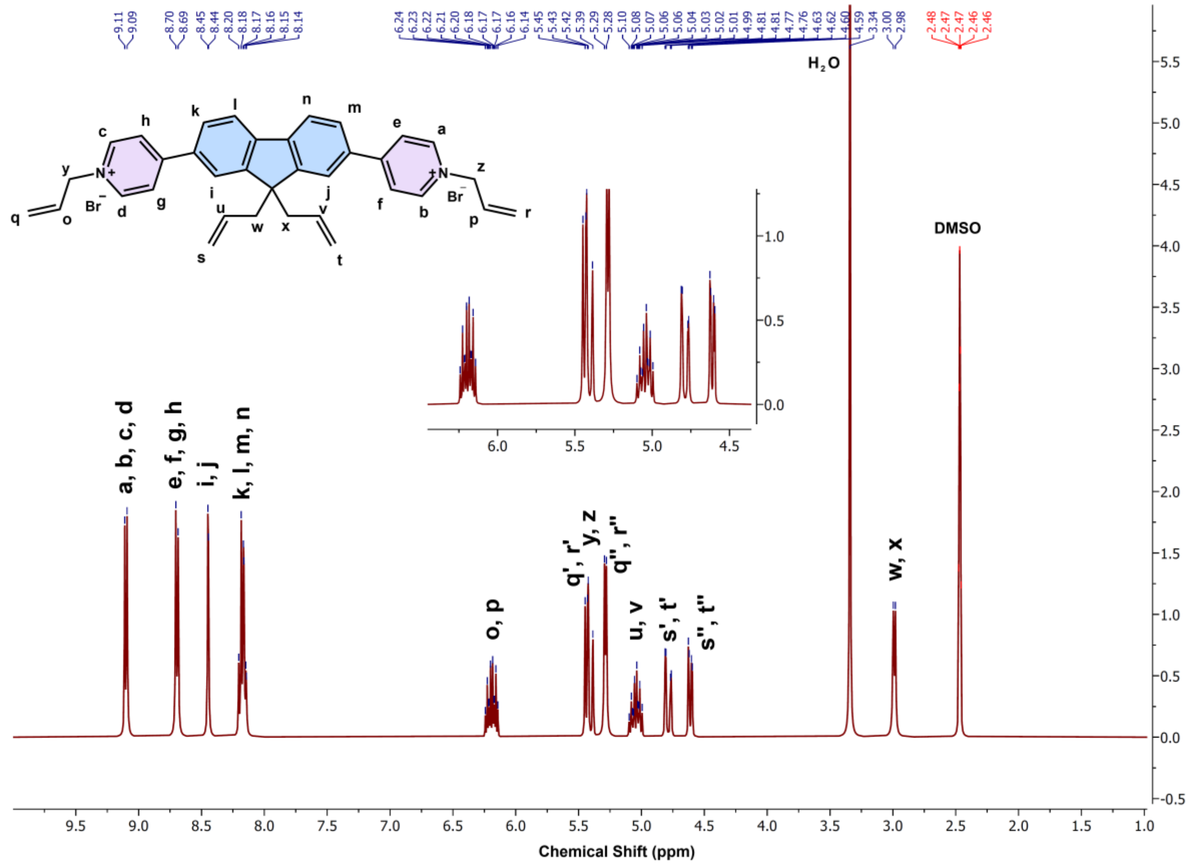

Figure S1. ^{1}H-NMR spectrum of FBP_allyl (solvent: DMSO-d_6).

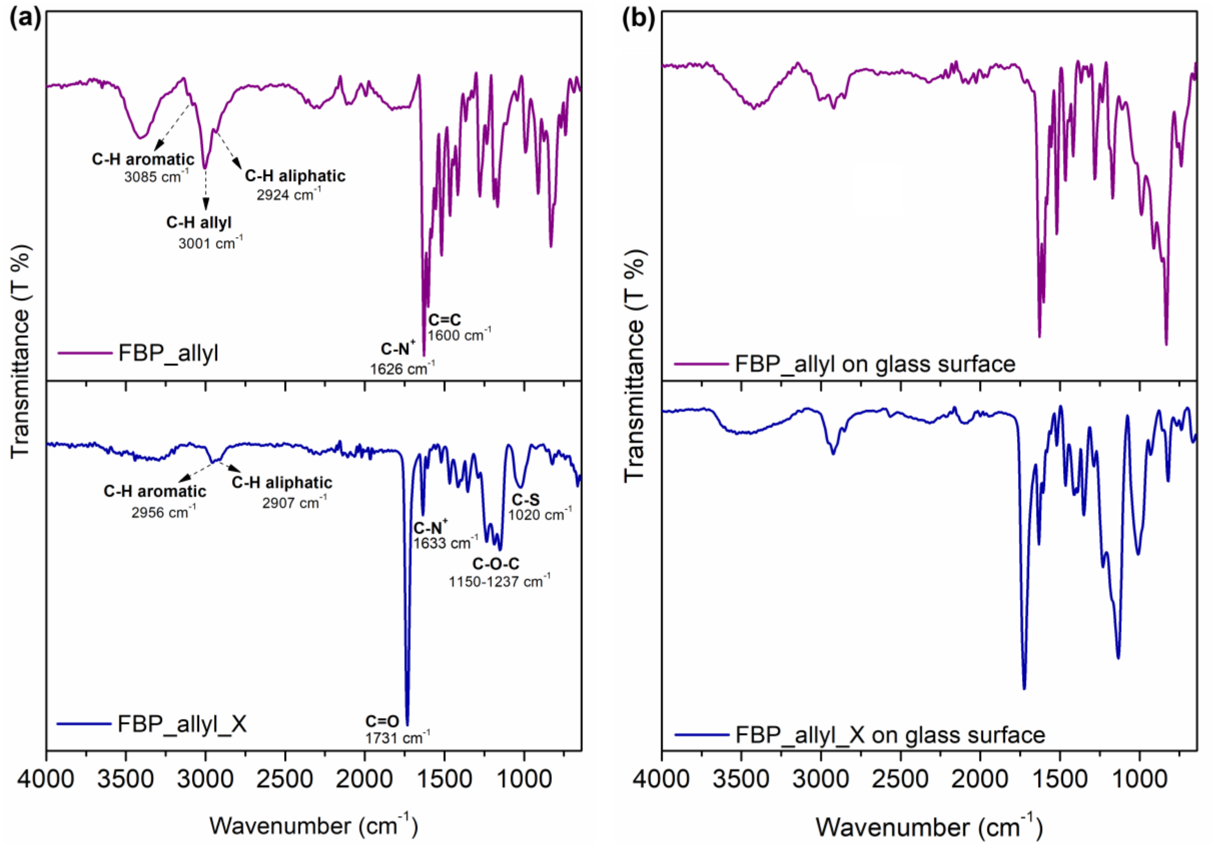

Figure S2. FT-IR spectra of (a) FBP_allyl and FBP_allyl_X in powder form and (b) FBP_allyl and FBP_allyl_X deposited on glass substrates.

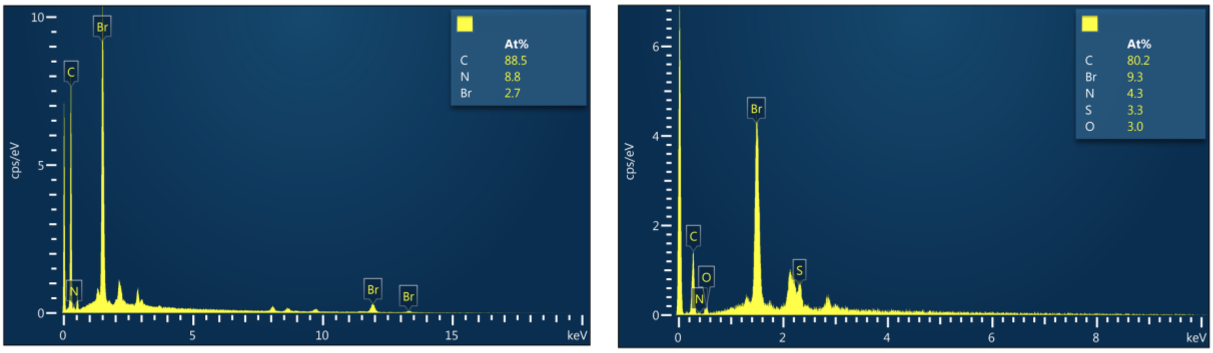

(a) (b)

Figure S3. SEM-EDX patterns of (a) FBP_allyl and (b) FBP_allyl_X electrodes.

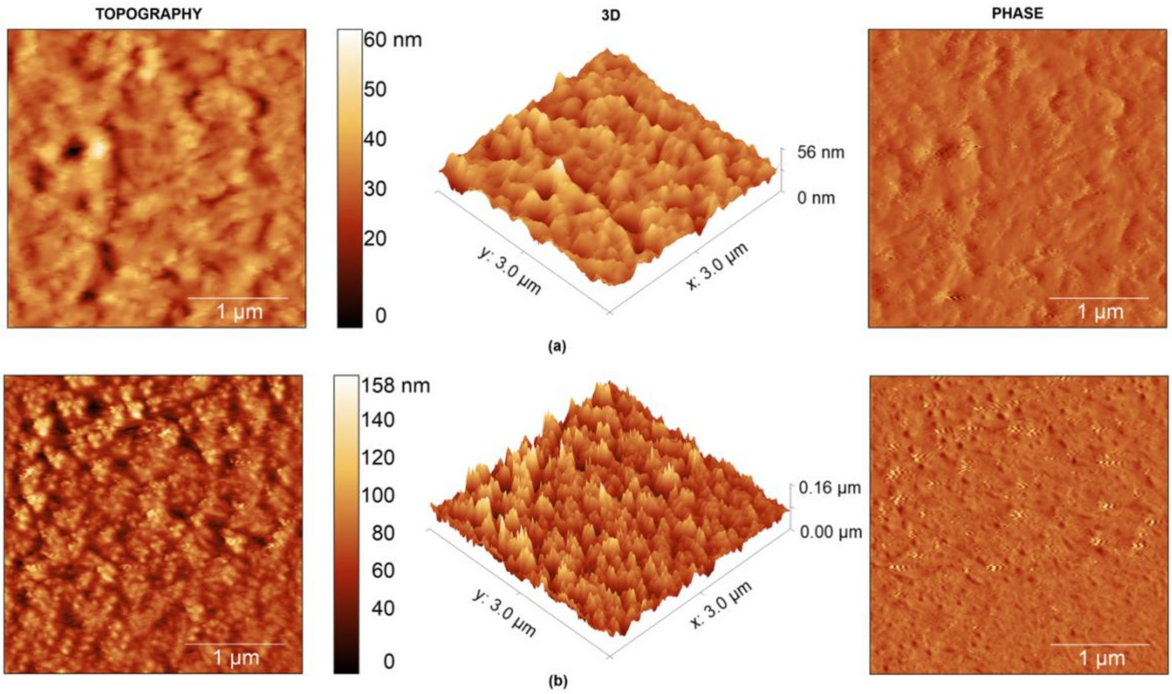

Figure S4. AFM images of (a) FBP_allyl and (b) FBP_allyl_X films on glass substrates.

Three-Electrode Photoelectrochemical Supercapacitor Assembly.

To assess the electrochemical energy-storage performance, a three-electrode photoelectrochemical supercapacitor was constructed with FBP_allyl_X as the photoactive electrode material. The working electrode consisted of a graphite sheet (effective area ~ 1 cm^2) coated with a cross-linked FBP_allyl_X polymer film. A platinum wire and an Ag/AgCl electrode served as the counter and reference electrodes, respectively. The electrodes were immersed in an aqueous 1 M H_2SO_4 electrolyte, providing a highly conductive and proton-rich medium for charge transport. Illumination was supplied by a visible-light Xe lamp (λ = 350-800 nm) focused directly on the FBP_allyl_X electrode surface, enabling evaluation under both dark and illuminated states. Electrical connections to the BioLogic SP-50e workstation were made via platinum clips to minimize contact resistance. Electrochemical characterizations included cyclic voltammetry (CV), galvanostatic charge-discharge (GCD), and electrochemical impedance spectroscopy (EIS, 100 kHz to 0.01 Hz, 10 mV amplitude). In addition, chronoamperometric and cycling stability tests were conducted under alternating light/dark conditions to probe the durability of the photoresponsive behavior.

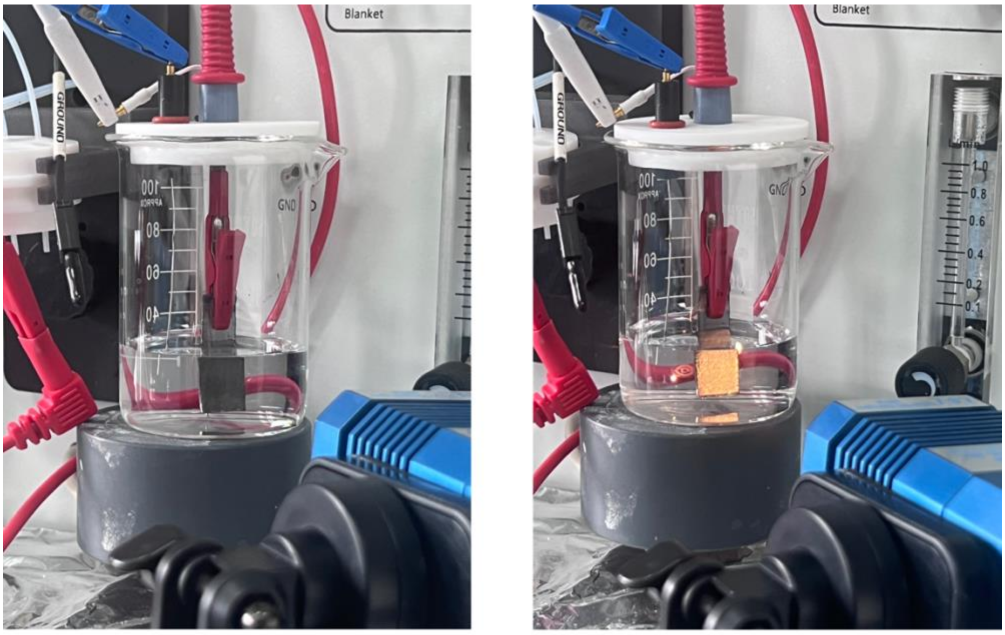

(a) (b)

Figure S5. Schematic illustration of photoelectrochemical supercapacitor (a) dark and (b) under illumination.

In the photoelectrochemical H-type supercapacitor system, the mass-specific capacity is calculated according to the GCD curve using the following equations:

$C_m = I \times t / m \times \Delta V$	(S-1)

where C_m is the gravimetric capacitance, m is the mass of active material (2 mg), I is the discharging current (A), t is the discharging time (s) and ΔV is the potential window. Electrode kinetic analysis is performed by the following equation:

$i = k_1\nu + k_2\nu^{1/2}$	(S-2)
$i/\nu^{1/2} = k_1\nu^{1/2} + k_2$	(S-3)

where i is absolute value of peak current density (A/g), ν is scan rate (mV/s).

The energy density and power density of the SC are calculated by the following equations:

$E = C_m \times \Delta V^2 / 7.2$	(S-4)
$P = E \times 3600 / t$	(S-5)

where E is the energy density and P is the power density.[8]

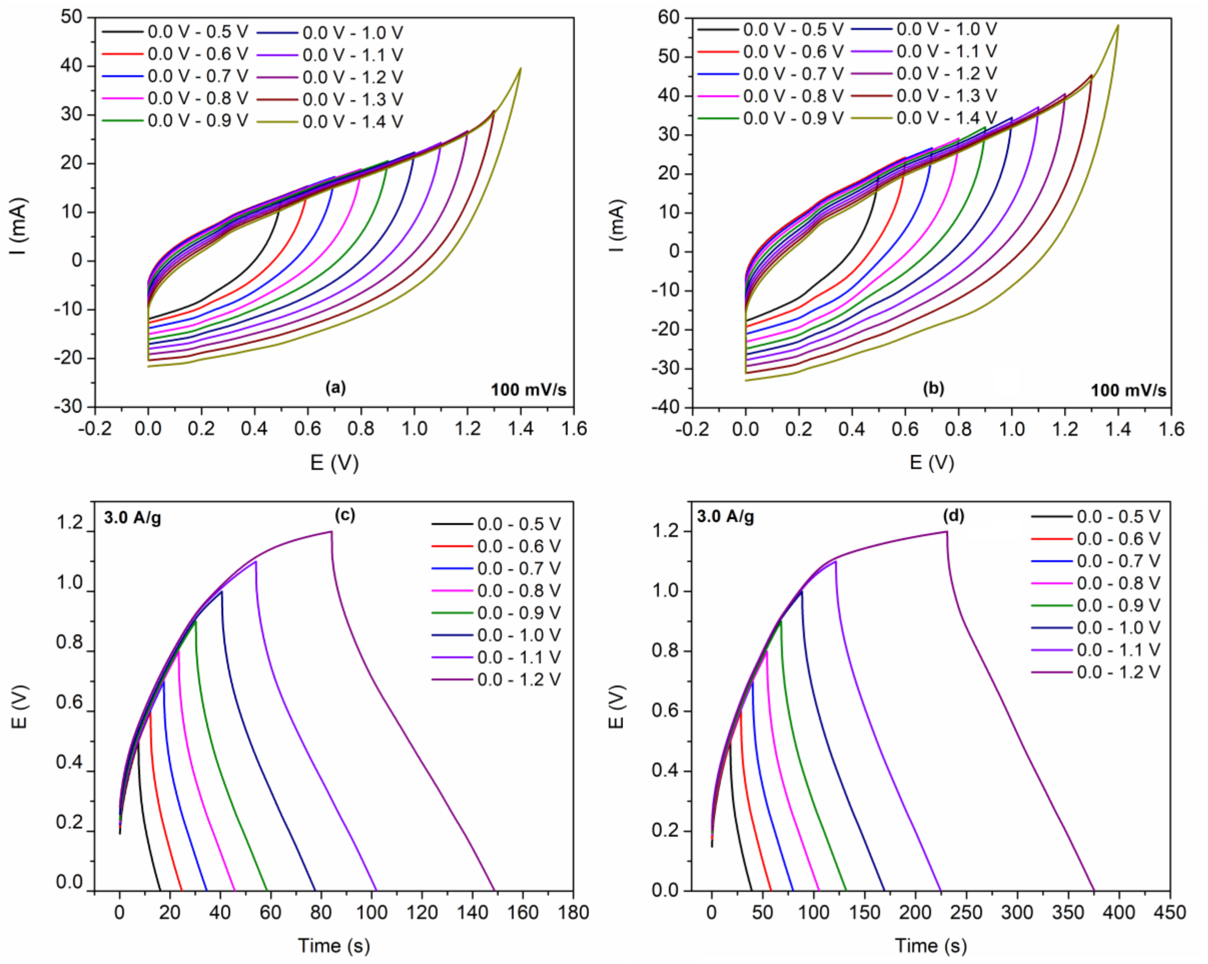

Figure S6. CV (a, b) and GCD (c, d) curves of the device under dark and illuminated conditions at different potential windows.

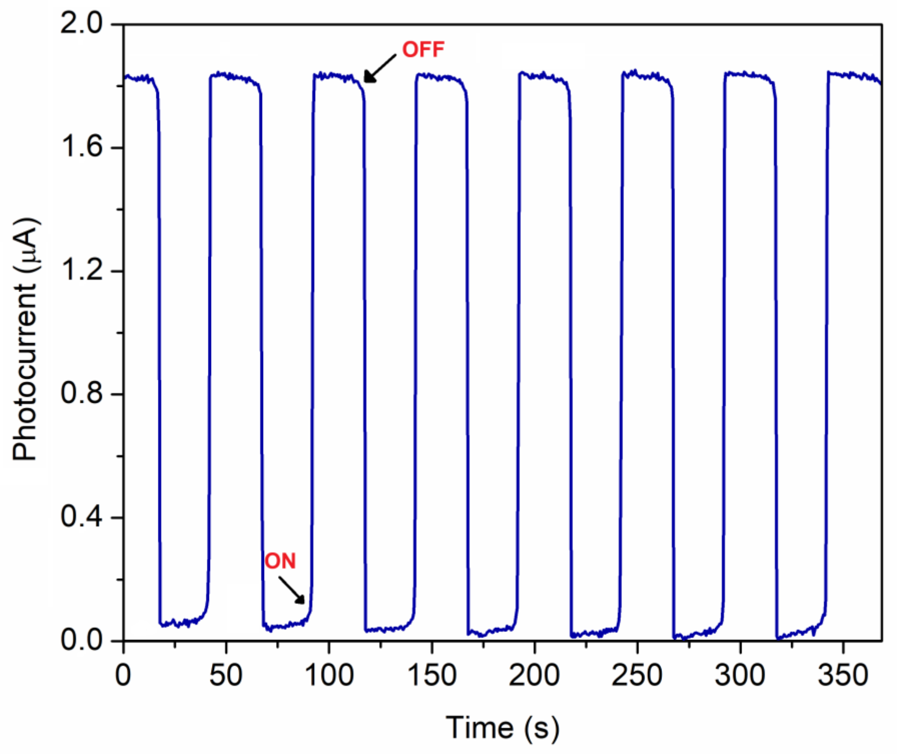

Figure S7. Photo-response of the FBP_allyl_X electrode under periodic on/off light illumination at zero applied bias (Ag/AgCl).

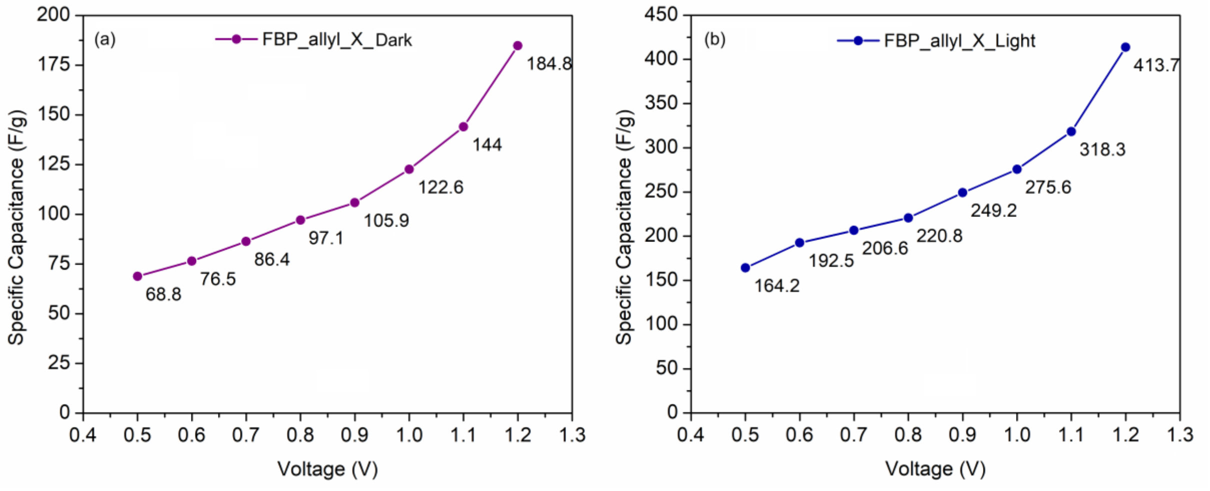

Figure S8. (a) Specific capacitance of FBP_allyl_X in the dark and (b) under illumination at different potential windows.

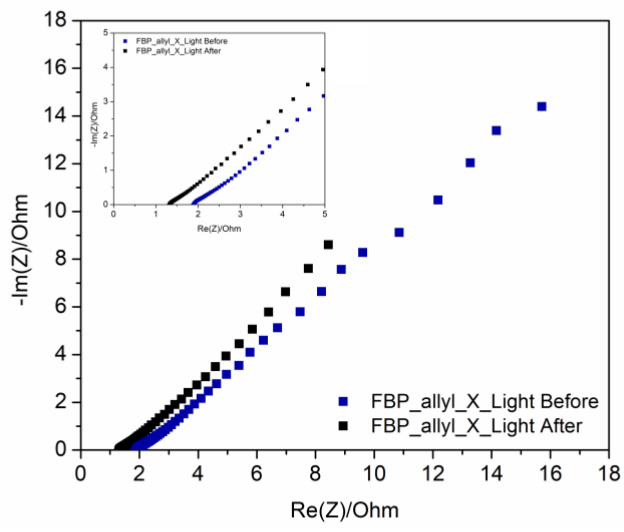

Figure S9. Nyquist plots of FBP_allyl_X electrode before and after the cyclic stability test.

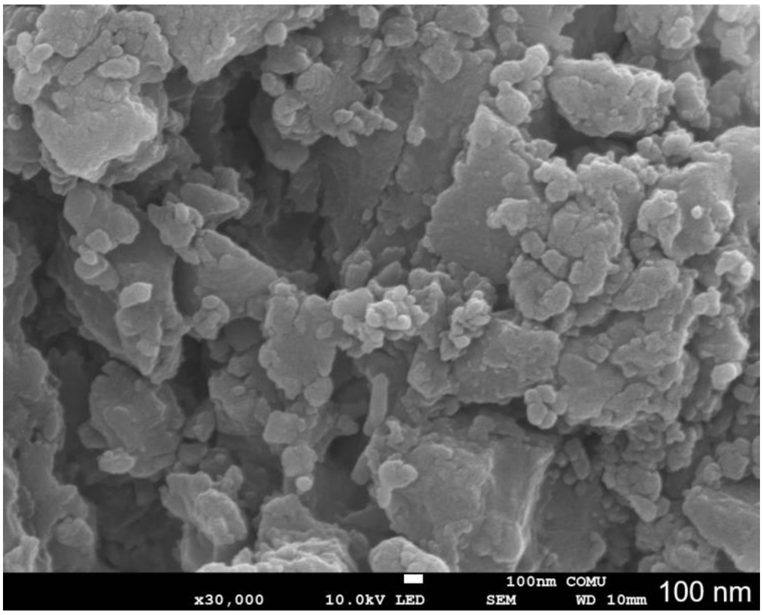

Figure S10. SEM images of FBP_allyl_X electrode after the cyclic stability test.

References

[1] Nakajo, T., Kumagai, J., Kusaka, S., Hori, A., Hijikata, Y., Pirillo, J., Ma, Y. & Matsuda, R. Triplet carbene with highly enhanced thermal stability in the nanospace of a metal–organic framework. *JACS*, **2021**, 143(21), 8129-8136.

[2] Jhaveri, S. B., Peterson, J. J., & Carter, K. R. Poly (9, 9-dihexylfluorene) layers grown via surface-directed Ni (0) condensation polymerization. *Langmuir*, **2009**, 25(16), 9552-9556.

[3] A.O. Patil, A.J. Heeger, F. Wudl, "Optical properties of conducting polymers" *Chem. Rev.* **1988**, 88, 1, 183-200.

[4] J. Heinze, B.A. Frontana-Uribe, S. Ludwigs, "Electrochemistry of Conducting Polymers-Persistent Models and New Concepts" *Chem. Rev.* **2010**, 110, 8, 4724-4771.

[5] M.J. Frisch, G.W. Trucks, H.B. Schlegel, G.E. Scuseria, M.A. Robb, J.R. Cheeseman, G. Scalmani, V. Barone, G.A. Petersson, H. Nakatsuji, X. Li, M. Caricato, A.V. Marenich, J. Bloino, B.G. Janesko, R. Gomperts, B. Mennucci, H.P. Hratchian, J.V. Ortiz, A.F. Izmaylov, J.L. Sonnenberg, Williams, F. Ding, F. Lipparini, F. Egidi, J. Goings, B. Peng, A. Petrone, T. Henderson, D. Ranasinghe, V.G. Zakrzewski, J. Gao, N. Rega, G. Zheng, W. Liang, M. Hada, M. Ehara, K. Toyota, R. Fukuda, J. Hasegawa, M. Ishida, T. Nakajima, Y. Honda, O. Kitao, H. Nakai, T. Vreven, K. Throssell, J.A. Montgomery Jr., J.E. Peralta, F. Ogliaro, M.J. Bearpark, J.J. Heyd, E.N. Brothers, K.N. Kudin, V.N. Staroverov, T.A. Keith, R. Kobayashi, J. Normand, K. Raghavachari, A.P. Rendell, J.C. Burant, S.S. Iyengar, J. Tomasi, M. Cossi, J.M. Millam, M. Klene, C. Adamo, R. Cammi, J.W. Ochterski, R.L. Martin, K. Morokuma, O. Farkas, J.B. Foresman, D.J. Fox, Gaussian 16 Rev. C.01, Wallingford, CT, 2016.

[6] R. Dennington, T.A. Keith, J.M. Millam, GaussView, version 6.0. 16, Semichem Inc Shawnee Mission KS, (2016).

[7] A.D. Becke, Density-functional exchange-energy approximation with correct asymptotic behavior, Physical review A, 38 (1988) 3098.

[8] B.H. Xiao, J.X. Li, H.Y. Xu, J.L. Huang, Y.L. Luo, K. Xiao, Z.Q. Liu, "Polymer Chainmail: Steric Hindrance and Charge Compensation of Anion-Doped PEDOT to Boost Stress Deformation of Compressible Supercapacitor" *Angew. Chem.* **2023** 135(39), e202309614.
